# Comparative Outcomes of Brachyury Vaccine vs. Imatinib in Advanced Chordoma: A Mayo Clinic Experience

**DOI:** 10.3390/cancers17213493

**Published:** 2025-10-30

**Authors:** Juan P. Navarro-Garcia de Llano, Harshvardhan G. Iyer, Harry C. Hoffman, Mahesh Seetharam, Steven Attia, Oluwaseun O. Akinduro

**Affiliations:** 1 Department of Neurosurgery, Mayo Clinic, Jacksonville, FL 32224, USA; navarrogarciadellano.juanpablo@mayo.edu (J.P.N.-G.d.L.); iyer.harshvardhan@mayo.edu (H.G.I.); hoffmahc@mail.uc.edu (H.C.H.); 2 Division of Hematology and Medical Oncology, Department of Medicine, Mayo Clinic, Phoenix, AZ 85054, USA; seetharam.mahesh@mayo.edu; 3 Department of Hematology and Oncology, Mayo Clinic, Jacksonville, FL 32224, USA; attia.steven@mayo.edu

**Keywords:** chordoma, bone neoplasms, imatinib, vaccines, brachyury

## Abstract

**Simple Summary:**

Chordoma is a rare, slow-growing cancer that affects the bones of the spine and skull base, with no FDA-approved therapies to date. Current therapies often provide limited benefit, and better options are needed. We compared two treatments: imatinib, a targeted therapy, and an experimental vaccine designed to stimulate the immune system. By reviewing data from patients at our institution, we evaluated their effectiveness and side effects. Our findings offer insight into the strengths and limits of current treatments and highlight the need for new approaches. This research provides a foundation for future studies to improve care and quality of life for patients with chordoma.

**Abstract:**

**Background/Objectives**: Chordoma, a rare slow-growing malignant bone tumor, remains therapeutically challenging due to its invasive nature. We examined institutional outcomes comparing imatinib and brachyury-directed vaccines. **Methods**: We used data from three sites of our quaternary care academic institution to analyze demographics, time to progression, overall survival, and adverse events in chordoma patients treated with imatinib or enrolled in a brachyury vaccine trial. **Results**: We included a total of 52 patients (8 in the brachyury vaccine cohort and 44 in the imatinib cohort) in the analysis. As expected, sacrococcygeal location was the most predominant in both cohorts: 62.5% in the brachyury cohort and 45.5% in the imatinib cohort. No demographic differences were present between the cohorts. ECOG was similar in both groups (*p* = 0.796). The primary outcome, time to progression (TTP), was shorter in the brachyury compared to the imatinib cohort (5.6 vs. 13 months, *p* = 0.0589), almost reaching statistical significance. Overall survival (OS) was comparable, 211 vs. 212 months for the brachyury and imatinib cohorts, respectively (*p* = 0.277). Although the brachyury vaccine cohort presented a higher incidence of adverse events than the imatinib cohort (75% vs. 31.8%, *p* = 0.021), the severity was milder. **Conclusions**: Imatinib showed longer disease control than the brachyury vaccine, though overall survival was similar. Future studies and deeper molecular insights are essential to develop better therapies and improve patient quality of life.

## 1. Introduction

Chordoma is a rare, slow-growing bone cancer believed to arise from embryologic remnants of the notochord within the vertebral bodies of the spine. While often described histologically as a low-grade tumor, their insidious course is often accompanied by local invasion and destruction, therapeutic resistance and tumor recurrence [1,2]. Overall survival of chordoma patients irrespective of age and sex in a comprehensive study of 400 cases from the Surveillance, Epidemiology, and End Results (SEER) database demonstrated an overall survival (OS) of 6.29 years [3].

Current standard of care (SOC) treatment for chordomas consists of surgical resection and adjuvant radiotherapy. The extent of surgical resection may be limited by the technical challenges and associated deficits of tumor removal in challenging areas such as the skull base and further correlate with a poorer prognosis. In a retrospective study of 195 clival chordoma patients, our group found that a resected tumor volume greater than 4.9 cm^3^ and an overall extent of resection equal or lower than 81.3% were independent predictors of tumor progression [4]. In addition to surgical resection, high-dose radiotherapy can be utilized for controlling sacrococcygeal chordoma progression whereas limitations for high-dose radiotherapy exist for cervical and skull base chordomas due to the radiosensitive nature and risks of demyelinating injury associated with nearby neurological tissue including the spinal cord, brainstem and cranial nerves [5,6]. Given the clinical challenges and therapeutic resistance of chordoma, recurrence rate is relatively high ranging from ~20 to 70% [7,8,9,10]. The addition of adjuvant proton therapy showed lower risk of recurrence compared to other non-proton radiation modalities [11].

Advanced chordomas have been reported to overexpress receptor tyrosine kinases (RTKs), including epidermal growth factor receptor (EGFR) [12] and activated platelet-derived growth factor receptor (PDGFRB) [13]. On this biologic basis, several tyrosine kinase inhibitors (TKIs) have been explored for aggressive or recurrent disease. Among them, imatinib, a PDGFR inhibitor first described in the late 1990s and approved by the U.S. Food and Drug Administration in 2001 for chronic myeloid leukemia [14], is a commonly used TKI in chordoma. Clinical practice typically selects patients with PDGFRB or PDGFB positivity, reflecting the intended target of the drug and aiming to enrich for benefit.

In 2012, Stacchiotti et al. reported results from a phase II trial that enrolled patients with advanced PDGFB- and/or PDGFRB-positive chordoma. Among 50 patients, the best response included one partial response (2%) and 35 cases of stable disease (70%), yielding a 64% clinical benefit rate [15]. Although objective responses were uncommon, the disease control observed supports the therapeutic rationale for imatinib in this population. These findings also underscore the heterogeneity of chordoma biology, where target expression may translate into disease stabilization rather than tumor shrinkage. As a result, ongoing strategies often consider imatinib within broader treatment sequences, including surgery and radiotherapy, and encourage biomarker-informed selection to optimize the likelihood of benefit. For this reason, alternative treatment options are greatly needed and currently under investigation.

First described by Virchow in 1857 as cartilage-derived, chordomas are now recognized as remnants of notochordal tissue. A key discovery in familial cases is the duplication of chromosome 6q27, which contains the transcription factor brachyury, essential for notochord development [16]. In sporadic chordomas, brachyury is uniquely overexpressed compared to other bone or cartilage tumors [17,18]. This pathognomonic feature provided a strong rationale for the development of vaccines designed to stimulate the immune system to target tumor cells expressing brachyury.

In the multicenter phase 2 BRACHY-CHOR-001 trial (ClincalTrials.gov NCT03595228), patients were primed with modified vaccinia Ankara-Bavarian Nordic (MVA-BN)-Brachyury and a single dose of fowlpox Virus (FPV)-Brachyury before starting radiation therapy (RT). After RT, they received FPV-Brachyury boosters every 4 weeks for 4 doses, then every 12 weeks thereafter. Although the trial did not reach its primary outcome, it showed partial responses and a progression-free survival (PFS) greater than 2 years in two out of twenty-six patients [19]. This trial included patients > 12 years of age with histologically confirmed metastatic or unresectable chordoma, planned RT to at least one target, and Eastern Cooperative Oncology Group (ECOG) performance status < 2 [19].

In this retrospective study conducted across the three sites of our quaternary care academic institution, we compared outcomes of patients with advanced chordoma treated with imatinib to those enrolled in the BRACHY-CHOR-001 trial at our institution.

## 2. Materials and Methods

We conducted a retrospective chart review of all patients diagnosed with chordoma whose records included notes referencing imatinib, Gleevec, brachyury, brachyury vaccine, or BRACHY-CHOR-001. This search was performed using our internal Mayo Data Explorer platform. We included patients of all ages across our three sites. Charts were independently reviewed to confirm histology, treatment exposures, and measurable disease per RECIST 1.1, and data were abstracted into a standardized form under IRB approval (16-009946 and 18-003951), with de-identified analyses across all sites.

A detailed review of each patient’s chart was carried out to extract the following variables: age, sex, race/ethnicity, comorbidities (including heart failure, atrial fibrillation, coronary artery disease, hypertension, diabetes, obesity, use of anticoagulation, history of deep vein thrombosis, and pulmonary embolism), body mass index (BMI), ECOG score, cancer-related pain, incontinence, primary chordoma location, metastatic disease before starting brachyury vaccine or imatinib, therapies received before starting brachyury vaccine or imatinib, adverse events (AEs) associated with either brachyury vaccine or imatinib, time from diagnosis to the start of imatinib or brachyury vaccine, time to progression (TTP, defined as time from initiation of treatment to disease progression as per RECIST 1.1 (Response Evaluation Criteria in Solid Tumors)), and overall survival (OS, defined as time from initial diagnosis to death from any cause).

Our primary outcome was TTP. Secondary outcomes included OS and adverse event incidence, assessed from therapy initiation and compared between treatment cohorts across timepoints.

Descriptive analyses of demographic and clinical variables were performed using GraphPad Prism (10.5). Continuous data is presented as medians with ranges. Categorical data is reported as frequencies and proportions. The Mann–Whitney U-test was utilized for non-parametric continuous variables. For categorical outcomes, the Chi-square test or Fisher’s exact test was applied. Kaplan–Meier curves were generated for both primary and secondary outcomes.

## 3. Results

A total of 52 patients were included in the analysis: 8 (15.4%) received the brachyury vaccine and 44 (84.6%) received imatinib. The median age was slightly higher in the brachyury cohort (68.5) compared to the imatinib cohort (62.5). Most patients were male in both cohorts (75% in the brachyury cohort vs. 68.2% in the imatinib cohort). All patients in the brachyury cohort were white, whereas the imatinib cohort included a more diverse population including 79.5% White, 9.1% Hispanic, 4.5% Asian, 4.5% Middle Eastern, and 2.3% with unknown ethnicity. The median BMI was in the brachyury cohort (29.1 kg/m^2^) vs. the imatinib cohort (25.8 kg/m^2^) (Table 1).

The most common comorbidities included essential hypertension (37.5% in brachyury vs. 52.3% in imatinib), coronary artery disease (25.0% vs. 9.1%), diabetes mellitus (12.5% vs. 11.4%), and obesity (50% vs. 27.3%). Only patients in the imatinib cohort had a history of congestive heart failure (11.4%), atrial fibrillation (11.4%), or stroke (11.4%). Anticoagulation use was reported in 50% of the brachyury cohort and 45.5% of the imatinib cohort.

Tumor location also varied between cohorts (Table 1). The most common location in both was the sacrococcygeal region, observed in 62.5% of the brachyury cohort and 45.5% of the imatinib cohort. Cervical spine involvement was seen in 25% of brachyury and 15.9% of imatinib patients, while lumbar tumors were found in 12.5% and 13.6%, respectively. Thoracic and skull base chordomas were only present in the imatinib cohort, occurring in 6.8% and 20.5% of patients, respectively.

As for the clinical picture before starting the brachyury vaccine or imatinib, the ECOG score was not statistically different between the two cohorts (*p* = 0.796). Nevertheless, the brachyury cohort had significantly more advanced disease than the imatinib cohort, with all patients having metastatic disease (100% vs. 61.4%, *p* = 0.042) (Table 1). Both groups underwent extensive therapeutic regimens before receiving either the brachyury vaccine or imatinib, including multiple surgical procedures, rounds of photon and proton radiotherapy, cryotherapy, and other targeted therapies. No statistically significant differences were detected between the cohorts (Table 2).

The incidence of adverse events was proportionally higher in the brachyury cohort compared to the imatinib cohort (75% vs. 31.8%, *p* = 0.021). The most common adverse events associated with the vaccine were chills and fatigue, whereas fatigue, nausea, diarrhea, hepatotoxicity; and muscle cramping predominated in the imatinib cohort, each occurring twice (Table 3). Despite limited detail for most AEs, the imatinib group had two events considered severe by the Common Terminology Criteria for Adverse Events (CTCAE) version 5.0: hepatotoxicity with AST 5.0–20.0 × ULN (grade 3) and febrile neutropenia complicated by pneumonia requiring hospitalization (grade 4).

Time from initial diagnosis to the start of systemic therapy (either brachyury vaccine or imatinib) differed between groups. The median time to treatment was longer in the brachyury group (94 months) compared to the imatinib group (41.5 months), with a hazard ratio of 2.27 (95% CI: 1.07–4.81) and a *p*-value of 0.4014. Although not statistically significant, this trend indicates that patients receiving the brachyury vaccine, initiated therapy later in their disease course.

**Figure 1:**
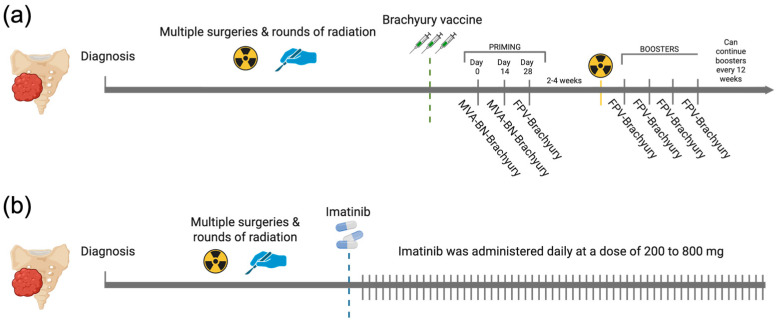
Overview of therapeutic regimens in the brachyury vaccine and imatinib cohorts. (**a**) Brachyury vaccine schedule showing two priming doses of MVA-BN-Brachyury plus a single FPV-Brachyury dose administered before RT, then FPV-Brachyury boosters every 4 weeks for four cycles and every 12 weeks thereafter. (**b**) Imatinib schedule showing continuous daily dosing at 200 to 800 mg. Created in BioRender. Navarro, J. (2025) https://BioRender.com/qn56xyi.

All patients in the brachyury cohort completed the full pre-RT regimen specified in the BRACHY-CHOR-001 trial (ClinicalTrials.gov NCT03595228), consisting of two priming doses of MVA-BN-Brachyury and one dose of FPV-Brachyury. In this cohort, the median (IQR) number of FPV-Brachyury boosters was 4 (4). In the imatinib cohort, the daily dose ranged from 200 to 800 mg, with most patients receiving 400 mg per day (61.35%) (Figure 1). The median duration of treatment was 158.5 (463.5) days for the brachyury cohort and 199.5 (234) days for the imatinib cohort.

**Figure 2:**
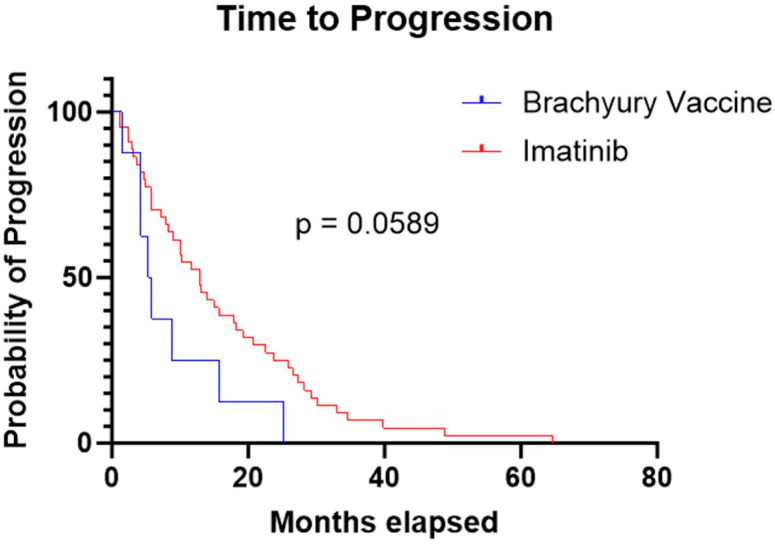
Kaplan–Meier curve demonstrating a longer time to progression in the imatinib cohort compared to the brachyury vaccine cohort (13 months vs. 5.6 months, respectively; *p* = 0.0589).

The median TTP was 5.6 months in the brachyury cohort and 13 months in the imatinib cohort. The log-rank (Mantel–Cox) test yielded a chi-square value of 3.567 with a *p*-value of 0.0589. The hazard ratio for progression was 0.43 (95% CI: 0.20–0.92), indicating a trend toward longer TTP period with imatinib, but this result was not statistically significant (Figure 2).

**Figure 3:**
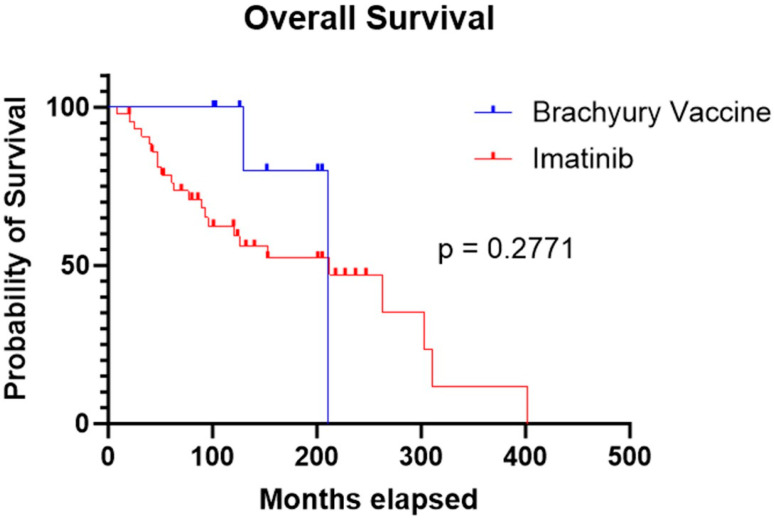
Kaplan–Meier curve showing similar OS in the imatinib cohort compared to the brachyury vaccine cohort (212 months vs. 211 months, respectively; *p* = 0.2771).

Median OS was comparable between the two cohorts: 211 months for patients treated with the brachyury vaccine and 212 months for those treated with imatinib. Statistical analysis showed no significant difference in survival between groups (*p* = 0.277) (Figure 3).

## 4. Discussion

In this study, we compared the outcomes of imatinib vs. brachyury vaccine for advanced chordomas. Although statistical significance was not reached (*p* = 0.059), likely due to the small sample size, imatinib showed a trend toward improved TTP after initiation of therapy when compared with the BRACHY-001 trial vaccines. However, there were no differences in overall survival (OS) between the two cohorts (*p* = 0.277), suggesting that while TTP improved with imatinib, the overall survival remained unchanged. Furthermore, although baseline clinical characteristics were not significantly different between the groups (*p* = 0.796), the unequal cohort sizes introduce a potential source of bias. In addition, the apparent advantage of imatinib over the brachyury vaccine may also be influenced by the fact that patients in the vaccine group typically received this therapy at a later stage following their initial diagnosis (median of 41.5 vs. 94 months, respectively; *p* = 0.4014). This longer interval aligns with the finding that all patients in the Brachyury cohort had metastatic disease at the time of vaccine administration (100%), compared with 61.4% in the imatinib group (*p* = 0.042), reflecting a more advanced disease course.

Another notable finding was that, despite a significantly higher proportion of adverse events in the brachyury vaccine cohort (*p* = 0.021), these were generally less severe (e.g., chills) compared with those in the imatinib cohort (e.g., febrile neutropenia, hepatotoxicity). The only two severe (CTCAE v5.0 ≥ 3) and potentially life-threatening adverse events were febrile neutropenia and hepatotoxicity, which occurred in patients receiving imatinib. However, the patient that presented febrile neutropenia was also on concomitant cisplatin, a drug known to cause myelosuppression [20], thereby limiting the validity of attributing this event solely to imatinib.

Imatinib is a selective tyrosine kinase inhibitor that works by competitively binding to the ATP-binding site of different receptors such as platelet-derived growth factor β (PDGFB), c-KIT, and BCR-ABL [21]. The presence of PDGFB and its ligands has been described previously in chordomas and has been correlated with a poor prognosis [22,23].

In 2012, Stacchiotti et al. published their phase II trial results evaluating the use of imatinib in 50 patients with advanced PDGFB-positive chordomas, showing a median PFS of 9 months [15]. Similarly, in 2015, Hindi et al. described a retrospective series of 48 patients showing a median PFS of 9.9 months [24]. In our series of 44 patients, we report a longer median TTP of 13 months, despite the similarities of their cohorts with ours both presenting the majority as sacrococcygeal and advanced disease. This difference could be potentially influenced by the different healthcare systems in which these cohorts were treated. We know that sociodemographic status plays an important role in the prognosis with this diagnosis [25].

As for AEs rates, the phase II trial reported a 72% incidence of grade ≥3 toxicities [15]. Although our observed incidence of adverse events was much lower, this difference is likely due to the more rigorous and systematic adverse event monitoring in a clinical trial compared with retrospective extraction from follow-up notes in routine practice. This same rationale may explain the disparity we observed between the brachyury and imatinib cohorts, given that the BRACHY-001 trial systematically recorded and reported all AEs.

The lack of improvement in clinical outcomes over the years remains a persistent challenge in the management of chordomas. Consequently, extensive research has focused on understanding the tumor’s biology to develop targeted therapies [26]. These efforts led to the discovery of brachyury as a unique diagnostic marker for chordomas, allowing their differentiation from chondrosarcomas and opening new avenues for therapeutic development [17,27,28].

Our institution participated in the BRACHY-CHOR-001 study, a phase 2 human trial in which patients with advanced chordomas received radiation in addition to a viral-based vaccine engineered to express the human brachyury tumor-associated antigen combined with human costimulatory molecules, aiming to maximize antitumor immune response [19]. The trial results showed that 2 (7.7%) of 26 patients achieved a partial response and PFS beyond two years and 8 (30.8%) showed disease stability and PFS greater than two years, remarkable findings in a disease with limited therapeutic options [19]. In our institutional cohort, the median TTP was 5.6 months. Consistent with published data, the vaccine’s safety profile was favorable, with no life-threatening adverse events reported.

The BN-Brachyury vaccine uses viral vectors to deliver the brachyury tumor antigen alongside three costimulatory molecules (B7.1, ICAM-1, LFA-3; TRICOM) that enhance T-cell activation. Infected antigen-presenting cells present brachyury-derived peptides on MHC molecules, while TRICOM strengthens immune synapse formation and provides potent secondary signals, driving robust expansion of brachyury-specific CD8^+^ cytotoxic T cells and supportive CD4^+^ T-cell responses [29]. The lack of improved outcomes with targeted therapies that have strong rationale, such as the brachyury vaccine, may be attributed to the well-documented tumor heterogeneity [30] and the complex immune microenvironment [31], which appears to play an immunosuppressive role against adaptive immunity. Studies have shown that most chordomas express PD-L1 [32], a central driver of immune evasion that could impair vaccine-induced T-cell responses. This highlights opportunities for combination strategies, such as checkpoint inhibition [33], to synergize with targeted therapies. However, this represents only part of the immunologic complexity; chordoma-secreted CCL5 has also been shown to promote macrophage recruitment and polarization toward an M2, tumor-supportive phenotype [34], adding another layer of complexity to the tumor’s immunoresistant biology.

Our comparison of these two experimental therapies suggests that, while one may appear to offer a modest advantage over the other, the ultimate survival outcomes are similar (OS of 211 vs. 212 months in the brachyury and imatinib cohorts, respectively). This underscores the ongoing need for research into the biological mechanisms of chordomas and their tumor microenvironment to guide more effective treatments. Given that surgery remains the cornerstone of chordoma management, this also presents an opportunity to explore local therapeutic delivery strategies (at the time of surgery), potentially overcoming barriers posed by the tumor’s hypoxic and poorly vascularized environment [35], which limits the efficacy of systemic drug administration.

As is well known for aggressive, treatment-resistant tumors, gene expression tends to “rewire” after recurrence, likely as a result of multiple rounds of surgery and/or radiation. For example, in 2021, Seeling et al. showed clearly different gene expression profiles in primary versus recurrent, same-patient–derived chordoma cell lines using whole-genome microarray analyses and identified the HOX/PBX complex as a potential target in recurrent or resistant chordomas [36]. In our study, both cohorts received treatment after multiple surgeries and radiation courses, which may influence responses to targeted therapies. How effective these therapies are in treatment-naive chordomas remains unclear, since the molecular dependencies seen after prior treatments may not be present before selection pressures act. Some targets may be less relevant in untreated tumors, while others could be more effective earlier, before compensatory pathways develop. Prospective studies that enroll treatment-naive patients, including baseline molecular profiling, and incorporate early on-treatment biopsies, are needed to determine the true benefit and optimal timing of targeted strategies in this setting.

This study has several limitations. First, its retrospective nature makes it subject to biases in medical record documentation and third-party interpretation, particularly in the reporting of AEs and clinical status (ECOG score, pain, etc.). Second, the sample size is small and not representative of the broader chordoma population, limiting the generalizability of our findings. In particular, the predominance of white male patients reflects a known limitation in chordoma research [37], where diversity remains insufficient for robust reproducibility. Larger, prospectively enrolled cohorts are still needed before drawing strong conclusions. Third, because treatment assignment was not randomized, differences in disease characteristics, comorbidities, or prior therapies between cohorts could have influenced the outcomes.

## 5. Conclusions

In this retrospective comparison, imatinib was associated with numerically improved disease control compared with brachyury vaccine, though overall survival was similar. It is important to note that patients receiving the brachyury vaccine began treatment at a more advanced disease stage (median of 94 vs. 41.5 months in imatinib, *p* = 0.4014). These findings underscore the need for prospective studies integrating molecular and immune biomarkers to optimize systemic therapy selection in advanced chordoma.

## Figures and Tables

**Table 1:** Characteristics of the cohorts imatinib vs. brachyury vaccine.

Variable	Brachyury Vaccine (N = 8)	Imatinib (N = 44)	*p* Value
Age (IQR)	68.5 (26)	62.5 (66)	0.449
Sex			0.701
Male	6 (75%)	30 (68.2%)	
Female	2 (25%)	14 (31.8%)	
Race/Ethnicity			0.739
Hispanic	0 (0%)	4 (9.1%)	
White	8 (100%)	35 (79.5%)	
Asian	0 (0%)	2 (4.5%)	
Middle Eastern	0 (0%)	2 (4.5%)	
Unknown	0 (0%)	1 (2.3%)	
Body Mass Index kg/m^2^ (IQR)	29.1 (12.6)	25.8 (25.5)	0.635
Essential Hypertension	3 (37.5%)	23 (52.3%)	0.442
Coronary Artery Disease	2 (25%)	4 (9.1%)	0.195
Congestive Heart Failure	0 (0%)	5 (11.4%)	0.316
Atrial Fibrillation	0 (0%)	5 (11.4%)	0.316
Stroke	0 (0%)	5 (11.4%)	0.316
Diabetes Mellitus	1 (12.5%)	5 (11.4%)	0.926
Obesity	4 (50%)	12 (27.3%)	0.200
Anticoagulation	4 (50%)	20 (45.5%)	0.813
Location			0.564
Skull Base	0 (0%)	9 (20.5%)	
Cervical	2 (25%)	7 (15.9%)	
Thoracic	0 (0%)	3 (6.8%)	
Lumbar	1 (12.5%)	6 (13.6%)	
Sacrococcygeal	5 (62.5%)	20 (45.5%)	
Disease Severity			
Pain	7 (87.5%)	33 (75%)	0.440
Motor Deficit	3 (37.5%)	18 (40.9%)	0.857
Sensory Deficit	4 (50%)	17 (38.6%)	0.547
Urinary Incontinence	2 (25%)	12 (27.3%)	0.894
Bowel Incontinence	1 (12.5%)	9 (20.5%)	0.599
ECOG			0.796
0	2 (25%)	11 (35.5%)	
1	6 (75%)	18 (58.1%)	
2	0 (0%)	1 (3.2%)	
3	0 (0%)	1 (3.2%)	
4	0 (0%)	0 (0%)	
Metastatic disease			0.042
Yes	8 (100%)	27 (61.4%)	
No	0 (0%)	17 (38.6%)	
Metastasis location			0.419
Local ^1^	2 (25%)	13 (48.2%)	
Distant ^2^	6 (75%)	14 (51.8%)	

^1^ Metastases to local soft tissues or osseous structures. ^2^ Distant metastases included lung, liver, and thyroid.

**Table 2:** Therapies received before brachyury vaccine or imatinib.

Variable	Brachyury Vaccine (N = 8)	Imatinib (N = 44)	*p* Value
Surgical Resection			0.999
Biopsy only	1 (12.5%)	7 (15.9%)	
One	2 (25%)	22 (50%)	
Two	3 (37.5%)	10 (22.7%)	
Three	2 (25%)	5 (11.4%)	
Radiation (photons)			0.249
No	2 (25%)	24 (54.5%)	
One round	3 (37.5%)	14 (31.8%)	
Two rounds	3 (37.5%)	4 (9.1%)	
Three rounds	0	2 (4.5)	
Radiation (protons)			0.443
No	4 (50%)	29 (65.9%)	
One round	3 (37.5%)	15 (34.1%)	
Two rounds	1 (12.5%)	0 (0%)	
Cryoablation			0.401
Yes	1 (12.5%)	2 (4.6%)	
No	7 (87.5%)	42 (95.5%)	
Other targeted therapies ^1^			0.130
Yes	3 (37.5%)	6 (13.6%)	
No	5 (62.5%)	38 (86.4%)	

^1^ Included immune checkpoint inhibitors (nivolumab and pembrolizumab), EGFR tyrosine-kinase inhibitor (erlotinib), multikinase/anti-angiogenic tyrosine-kinase inhibitors (pazopanib and sorafenib), BCR-ABL/c-KIT/PDGFR tyrosine-kinase inhibitor (nilotinib), antimetabolite (pemetrexed), and akylating agent (temozolomide).

**Table 3:** Adverse Events.

Variable	Brachyury Vaccine (N = 8)	Imatinib (N = 44)	*p* Value
Adverse Events	6 (75%)	14 (31.8%)	0.021
Chills	4	0	
Fatigue	3	2	
Fever	1	0	
Headache	1	1	
Rash	1	1	
Pruritus	1	1	
Nausea	0	2	
Diarrhea	0	2	
Hepatotoxicity ^1^	0	2	
Muscle Cramping	0	2	
Febrile neutropenia ^2^	0	1	
Neck fasciculations	0	1	
Anasarca	0	1	
Lower Extremity Edema	0	1	

^1^ Elevation AST above the normal range, ^2^ Patient was on concomitant cisplatin.

## Data Availability

Due to patient privacy and institutional policy, the underlying data from this retrospective study are not publicly available. De-identified data may be shared upon reasonable request to the corresponding author and with approval from the Institutional Review Board and a data use agreement.

## Abbreviations

The following abbreviations are used in this manuscript:

ECOG	Eastern Cooperative Oncology Group
TTP	Time to progression
OS	Overall survival
SOC	Standard of care
TKI	Tyrosine kinase inhibitor
MVA-BN	modified vaccinia Ankara-Bavarian Nordic
FPV	fowlpox Virus
RT	Radiation therapy
PFS	Progression free survival
BMI	Body mass index
PDGFB	platelet-derived growth factor β
